# Application of the Thermal Analysis of Frozen Aqueous Solutions to Assess the Miscibility of Hyaluronic Acid and Polymers Used for Dissolving Microneedles

**DOI:** 10.3390/pharmaceutics16101280

**Published:** 2024-09-30

**Authors:** Ken-ichi Izutsu, Hiroyuki Yoshida, Yasuhiro Abe, Eiichi Yamamoto, Yoji Sato, Daisuke Ando

**Affiliations:** 1 School of Pharmacy at Narita, International University of Health and Welfare, Kozunomori 4-3, Narita 286-8686, Japan; 2 Division of Drugs, National Institute of Health Sciences, Tonomachi 3-25-26, Kawasaki 210-9501, Japan; h.yoshida@nihs.go.jp (H.Y.); abey@nihs.go.jp (Y.A.); yoji@nihs.go.jp (Y.S.); ando-daisuke@nihs.go.jp (D.A.); 3 Division of Medical Devices, National Institute of Health Sciences, Tonomachi 3-25-26, Kawasaki 210-9501, Japan; eyamamoto@nihs.go.jp

**Keywords:** hyaluronic acid, phase separation, freeze concentration, drug delivery matrix, microneedle

## Abstract

**Background:** The combination of multiple polymers is anticipated to serve as a means to diversify the physical properties and functionalities of dissolving microneedles. The mixing state of components is considered as a crucial factor in determining their suitability. **Objectives:** The purpose of this study was to elucidate whether thermal analysis of frozen aqueous solutions can appropriately predict the miscibility of hyaluronic acid (HA) and other polymers used for dissolving microneedles prepared by a micromolding method. **Methods:** Aliquots of aqueous polymer solutions were applied for thermal analysis by heating the samples from −70 °C at 5 °C/min to obtain the transition temperature of amorphous polymers and/or the crystallization/melting peaks of polymers (e.g., polyethylene glycol (PEG)). Films and dissolving microneedles were prepared by air-drying of the aqueous polymer solutions to assess the polymer miscibility in the solids. **Results:** The frozen aqueous single-solute HA solutions exhibited a clear T_g_′ (the glass transition temperature of maximally freeze-concentrated solutes) at approximately −20 °C. The combination of HA with several polymers (e.g., dextran FP40, DEAE-dextran, dextran sulfate, and gelatin) showed a single T_g_′ transition at temperatures that shifted according to their mass ratio, which strongly suggested the mixing of the freeze-concentrated solutes. By contrast, the observation of two T_g_′ transitions in a scan strongly suggested the separation of HA and polyvinylpyrrolidone (PVP) or HA and polyacrylic acid (PAA) into different freeze-concentrated phases, each of which was rich in an amorphous polymer. The combination of HA and PEG exhibited the individual physical changes of the polymers. The polymer combinations that showed phase separation in the frozen solution formed opaque films and microneedles upon their preparation by air-drying. Coacervation occurring in certain polymer combinations was also suggested as a factor contributing to the formation of cloudy films. **Conclusions:** Freezing aqueous polymer solutions creates a highly concentrated polymer environment that mimics the matrix of dissolving microneedles prepared through air drying. This study demonstrated that thermal analysis of the frozen solution offers insights into the mixing state of condensed polymers, which can be useful for predicting the physical properties of microneedles.

## 1. Introduction

Application of hyaluronic acid (HA) for dissolving microneedles is attracting increasing attention because of its superior biocompatibility and biodegradability, low toxicity, and hydrophilic character [1,2,3,4,5,6,7,8,9]. Developing microneedle products that satisfy the desired physical properties and performance often requires the careful optimization of the formulation components [5,10,11,12,13,14,15]. In addition to the choice of appropriately sized HA molecules, their combination with several other polymers or low-molecular-weight excipients (e.g., disaccharides) is a popular approach for achieving the desired properties of the HA-based microneedles and other delivery matrices [5,7,12,13,14,15,16,17,18,19,20,21,22,23,24,25].

The mixing states of components are considerable factors that determine the physical properties and pharmaceutical performance of HA-based polymer composites. Some formulation processes that are used to concentrate the aqueous solutions often induce phase separation in the polymer combinations, while others remain mixed during the same process [26,27,28,29,30,31,32]. Various factors inherent to the formulations (e.g., polymer combinations, mass ratio, and co-solute composition) and processes (e.g., drying methods) may affect the miscibility of the polymers [33,34]. The phase separation of HA and PEG is a useful method for creating macroporous hydrogels [35]. Moreover, some polymers tend to crystallize in phase-separated environments.

Information on the mixing state and crystallinity of the components of the matrices should be relevant in the rational design of HA-based dissolving microneedles. However, assessing the mixing state of the non-crystalline components of the solid formulations remains challenging. The popular methods that are used to study the mixing state of the non-crystalline multicomponent solids, including thermal analysis (e.g., the profile of the glass transition temperature (T_g_)), spectroscopy mapping (e.g., Raman and IR), and visual observation, do not always provide sufficient information [36,37]. The analysis of the interactions between the molecules in the solutions (e.g., turbidimetric titration, confocal scanning laser microscopy, dynamic light scattering, and zeta potentiometry) helps predict their miscibility in solids; however, its use remains limited [38,39]. The high intrinsic propensity of HA to be dried as an amorphous solid and the possible immiscibility of the polymers in the condensed states emphasize the importance of improving or identifying methods to evaluate their mixing states [40]. Some components tend to crystallize after their phase separation, providing a better chance of detection using several methods, including powder X-ray diffractometry (PXRD), thermal analysis, and several spectroscopic mapping methods [41].

This study aimed to clarify the mixing states of HA and other polymers in frozen solutions using thermal analysis and to assess the applicability of the method to the design of dissolving microneedles and other drug delivery matrices. The freezing of aqueous solutions highly concentrates (e.g., above 70%, *w*/*w*) the solutes into a narrow phase surrounding ice crystals until the increasing local viscosity perturbs further ice growth [42]. The increasing concentration obtained by ice growth induces the phase separation of some polymer combinations via the exact same mechanism of liquid–liquid phase separation, whereas other solutes are concentrated into their mixture phase [43]. Various polymers and low-molecular-weight excipients have an intrinsic “glass transition temperature of the maximally freeze-concentrated solute (T_g_′)” of the frozen solution, above which the viscosity of the freeze-concentrated considerably decreases [44,45,46]. Thus the number and temperature profiles of the T_g_′ transitions, which are obtained via the thermal analysis of the multi-solute frozen solutions, would provide valuable information regarding the mixing states of the non-crystalline concentrated polymers [43]. The removal of ice by sublimation (freeze-drying) retains the varied mixing states of the polymers during the process [37,47]. In addition, the thermal analysis of frozen solutions indicates the crystallization and crystal melting of certain solutes [34]. It is of particular interest to determine whether thermal analysis of frozen solutions, which mimics the dehydration process, can be used to estimate the mixing state of polymers in HA-based microneedles and other drug delivery matrices formed by drying aqueous solutions [31].

## 2. Materials and Methods

### 2.1. Materials

Sodium hyaluronate of several sizes manufactured via a fermentation process using *Streptococcus zooepidemicus* (FCH-SU: 50–110 kDa, FCH-60: 500–700 kDa, FCH-80: 600–1000 kDa, FCH-120: 1000–1400 kDa) was purchased from Kikkoman Biochemifa Co. (Tokyo, Japan). Sucrose, D-(+)-glucose, dextran sulfate sodium salt (molecular weight (MW), 7000–20,000), dextran sulfate sodium salt (average MW, 5000), diethylaminoethyl (DEAE)-dextran hydrochloride, DEAE-dextran (average MW, 10,000), poly(acrylic acid sodium salt) (average MW, <8000), polyvinylpyrrolidone (PVP) (average MW, 10,000, 29,000, and 40,000), poly(vinyl alcohol) (MW, 9000–10,000, 80% hydrolyzed), and carboxymethylcellulose sodium salt (low viscosity) were obtained from Sigma-Aldrich Co.(St. Louis, MO, USA). Other chemicals were obtained from FUJIFILM Wako Pure Chemicals Co. (polyethylene glycol (PEG) 6000: MW, 7300–9300, Tokyo, Japan), SERVA Electrophoresis GmbH (dextran 4: MW, 3500–7500; dextran FP40: MW, 35,000–45,000, Heidelberg, Germany), Fisher Scientific (PVP: average MW, 3500, Pittsburgh, PA, USA), KIMICA Co. (sodium alginate, 15–25 cps, Tokyo, Japan), Nippi Co. (gelatin type AP: bovine skin; average MW, 8000, Tokyo, Japan), and Polysciences Inc. (cellulose, hydroxyethyl ether: MW, <90,000, HEC, Warrington, PA, USA). All chemicals were used without further purification. In the following text, sodium hyaluronate and hyaluronic acid are abbreviated as HA, unless otherwise specified.

### 2.2. Thermal Analysis of Frozen Solutions and Solids

Thermal analyses of frozen aqueous solutions were conducted using a differential scanning calorimeter (DSC-Q2000, TA Instruments, New Castle, DE, USA). The aliquots (20 μL) of solutions placed in hermetic aluminum cells were scanned by heating from −70 °C at 5 °C/min. After the initial scans were paused at −5 °C and maintained at that temperature for 1 min, the samples were cooled again to −70 °C for the second heating scans to obtain thermal transitions. The data from the thermal analysis were processed using a software (Universal Analysis 2000, TA Instruments). Solids containing different mass ratios of HA and PEG were obtained by drying aliquots of the aqueous solutions (20 μL) in open aluminum DSC cells in an atmospheric pressure chamber for 1 day and then under reduced pressure overnight at room temperature (approx. 25 °C). Hermetically sealed aluminum cells were scanned by heating from −10 °C at 10 °C/min under nitrogen gas flow.

### 2.3. Preparation of Polymer Films and Dissolving Microneedles

Aqueous polymer solutions (140 μL) were poured into circular spaces (diameter, 10 mm) to form films at room temperature without centrifugation (thickness, approx. 0.5 mm), as described in previous paper [48]. The solutions were dried at room temperature overnight in the presence of silica gel. Dissolving microneedles composing HA as base material were fabricated using the micromolding method via the air-drying of aqueous solutions (cone-shaped needle; height: 600 μm; base diameter: 300 μm; interspacing of needles: 600 μm; density: 121/patch) [49]. In short, aqueous polymer solutions were cast onto polydimethylsiloxane (PDMS) molds before their centrifugation at 2380× *g* for 15 min to remove air bubbles and fill the holes. Then, the samples were dried in an oven at 25 °C with dry silica gel. The presence of phase separation was evaluated based on the appearance, such as cloudiness, in the films and microneedles prepared by drying.

## 3. Results

### 3.1. Properties of Single-Solute Frozen Polymer Solutions

The thermal analysis of frozen HA solutions revealed a clear thermal transition in the heating scans, indicating the non-crystalline state of the freeze-concentrated polymer. HAs with varying MWs (sodium hyaluronates: FCH-SU, FCH-60, FCH-80, FCH-120, 1%, *w*/*w*) indicated the T_g_′ of the frozen solutions at approximately −20 °C (Table 1). Increasing the HA concentration (FCH-SU, 1–6%, *w*/*w*) did not apparently alter the transition temperature. The second scan of the frozen solution after the first scan of up to −5 °C slightly increased the transition temperatures. A lower-MW HA (FCH-SU) was used in subsequent experiments because of the lower viscosity of its solutions.

**Figure 1:**
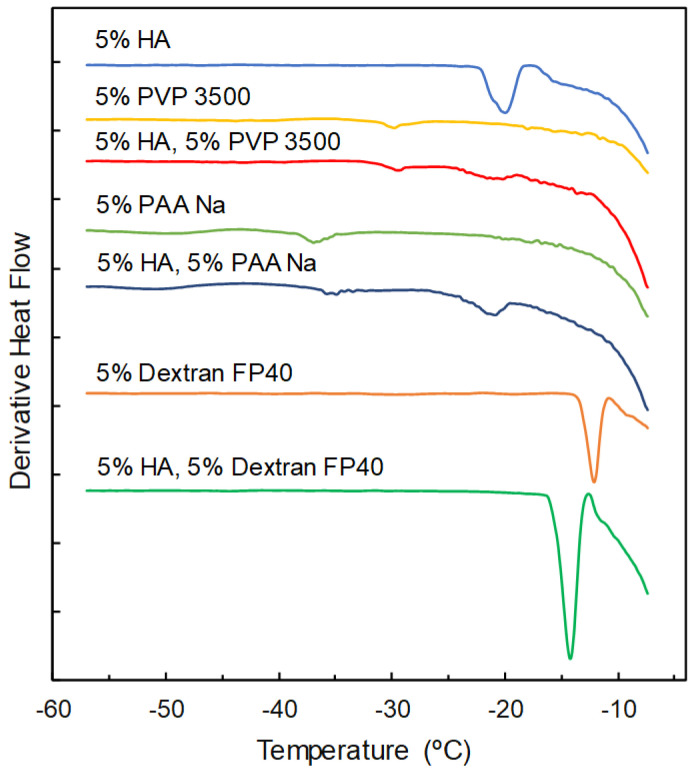
Derivative heat flow curves of frozen solutions comprising hyaluronic acid (HA), polyvinylpyrrolidone (PVP) 3500, dextran FP40, PAA Na, and their mixtures obtained through heating scans (20 μL, 5 °C/min).

The single-solute frozen polymer and disaccharide solutions exhibited heat flow curves that were categorized into three groups. Some solutes (e.g., dextran 4, dextran FP40, PVP 3500, PVP 10,000, PVP 29,000, PVP 40,000, PAA Na, DEAE-dextran 10,000, DEAE-dextran HCl, dextran sulfate Na, gelatin, glucose, and sucrose) showed an apparent T_g_′ transition that indicated the amorphous state of the freeze-concentrated solutes (Table 2). Increasing the MW of dextran, PVP, or dextran sulfate Na shifted the transition to higher temperatures [46]. Thermal transition (T_g_′) was observed as peaks in the derivative heat flow curves (Figure 1). Some solutes displayed crystallization (exothermic; PEG and mannitol) or melting (endothermic; PEG) peaks of the eutectic crystal in thermal analysis. A heating scan of the frozen solutions comprising a third group of solutes (e.g., glycine, PVA 10,000, sodium alginate, CMC Na, and HEC) showed only the large endotherm of ice melting at around 0 °C. Some of these solutes may have crystallized during the cooling process of the solutions.

### 3.2. Mixing States of Freeze-Concentrated HA and Polymer Combinations

Frozen solutions comprising HA and co-solutes exhibited their different mixing states in the freeze-concentrated region. The single obvious transition of the HA and dextran FP40 combination frozen solution, which was observed as a peak in the derivative heat flow, detected at the temperatures between those of the single components indicated the mixing of the polymers in the freeze-concentrate (Figure 1). The combination of HA and PVP 3500 or PAA Na showed two transitions at temperatures that were close to those of the single-solute solutions of the components, suggesting their separation into phases, each of which was rich in one of the polymers.

**Figure 2:**
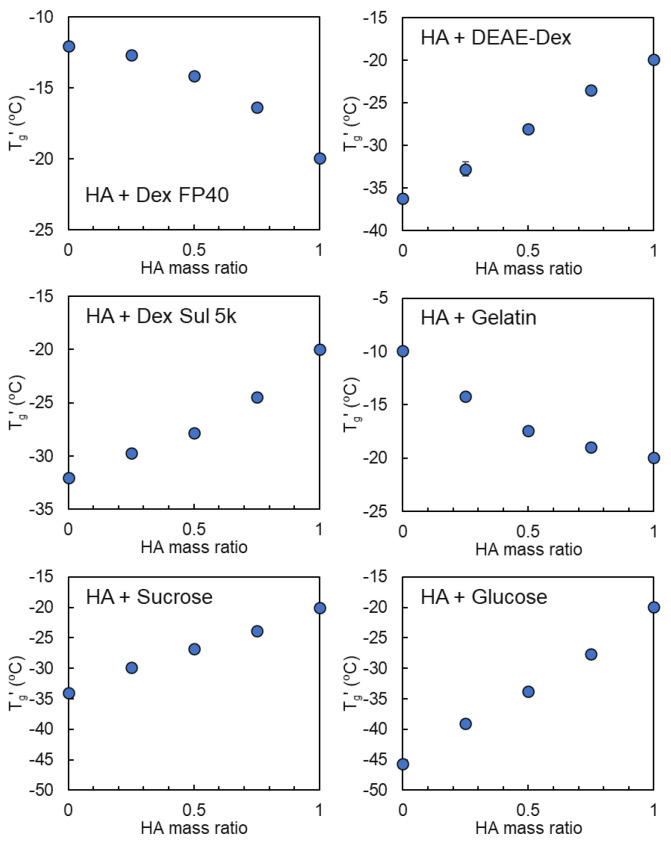
Transition temperature (T_g_′) profiles of the frozen solutions comprising hyaluronic acid (HA) and co-solutes showing a continuous change according to the solute mass ratios (6% total, n = 3, average ± SD). The symbols denote the temperature of the first (◯) transitions in the heating scan.

**Figure 3:**
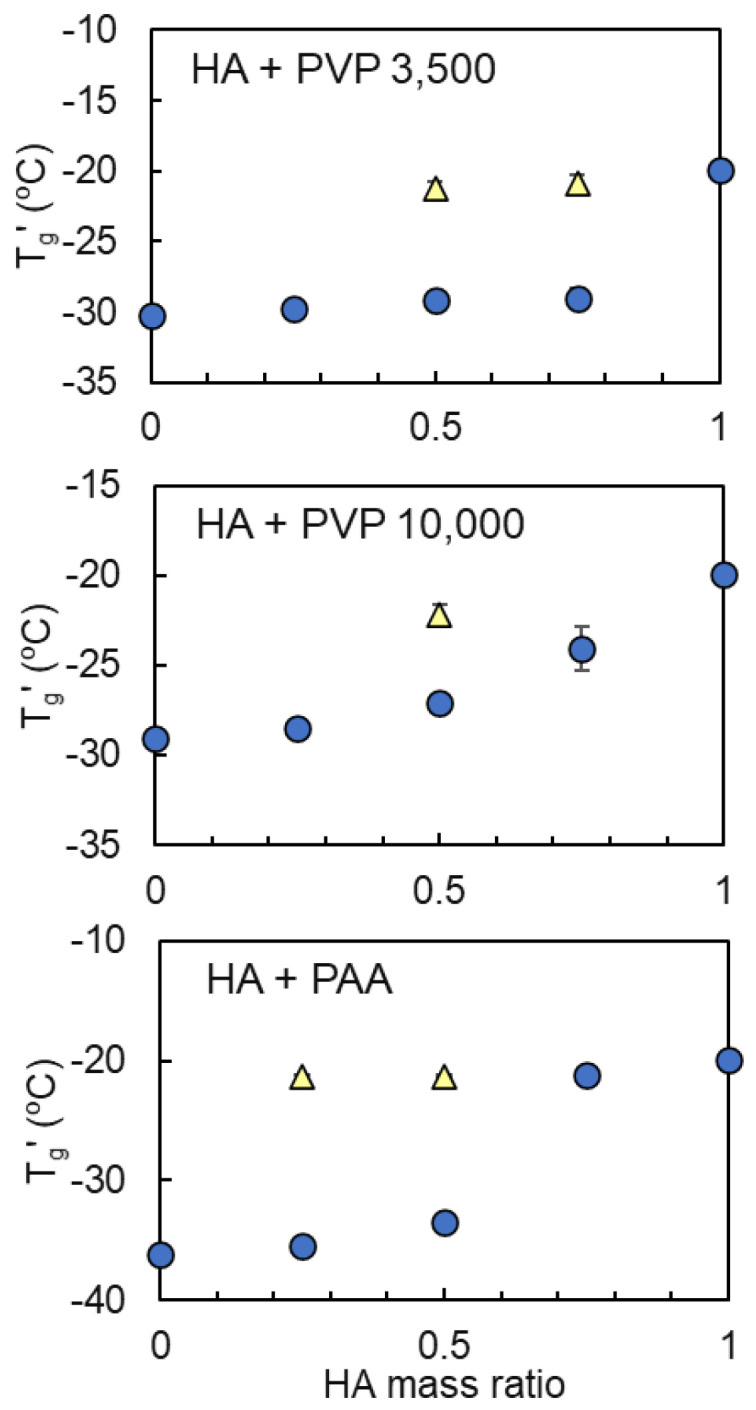
Transition temperature (T_g_′) profiles of the frozen solutions comprising hyaluronic acid (HA) and co-solutes showing two transitions at some solute mass ratios (6% total, n = 3, average ± SD). The symbols denote the temperature of the first (◯) and second (△) transitions in the heating scan, respectively.

**Figure 4:**
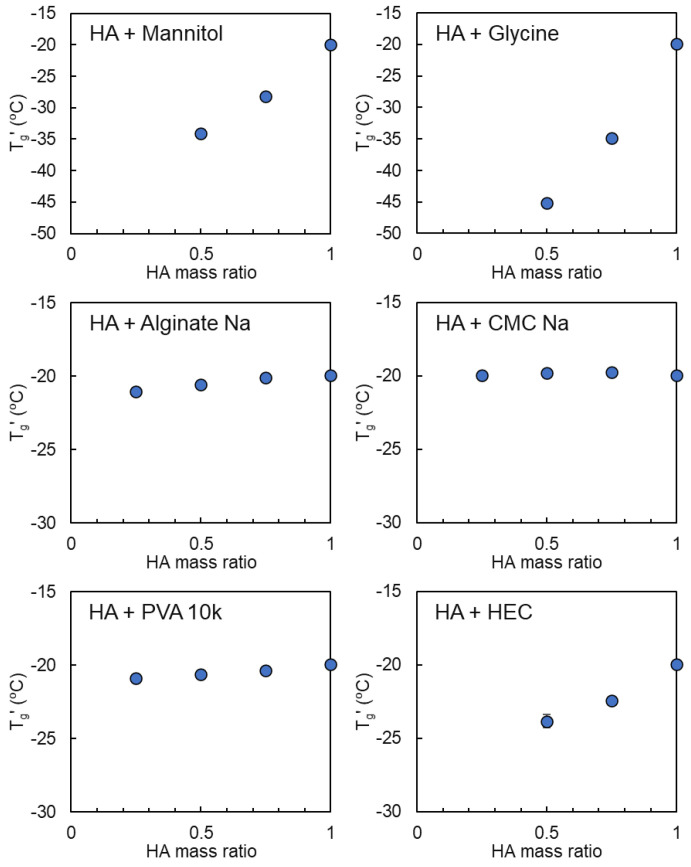
Transition temperature (T_g_′) profiles of the frozen solutions comprising hyaluronic acid (HA) and co-solutes that did not show an obvious transition in the single-solute frozen solutions (6% total, n = 3, average ± SD). The symbols denote the temperature of the first (◯) transitions in the heating scan.

The transition temperatures of the frozen solutions comprising HA and co-solutes at several mass ratios are shown in Figure 2, Figure 3 and Figure 4. The measurements were conducted with a total polymer concentration of 6% in each solution. The combination of HA with some polymers or saccharides yielded an apparent transition at temperatures that shifted between the intrinsic T_g_′ of the components, indicating the mixing of the concentrated non-crystalline HA and various solutes (dextran FP40, DEAE-dextran 10,000, dextran sulfate Na 5000, gelatin, glucose, and sucrose) in the non-ice region (Figure 2). The combination of HA and DEAE-dextran HCl was not applied for thermal analysis because of the occurrence of obvious clouding upon mixing, presumably by the complex coacervation of HA with cationic polymer [50]. The attractive interaction between HA and several cationic polymers often induces the coacervation in the mixed solutions [51]. By contrast, frozen solutions comprising HA and another group of polymers (PVP 3500, PVP 10,000, and PAA Na) exhibited two transitions at certain mass ratios, which suggested the separation of HA and these polymers into different freeze-concentrated phases that were rich in one of the components (Figure 3).

**Figure 5:**
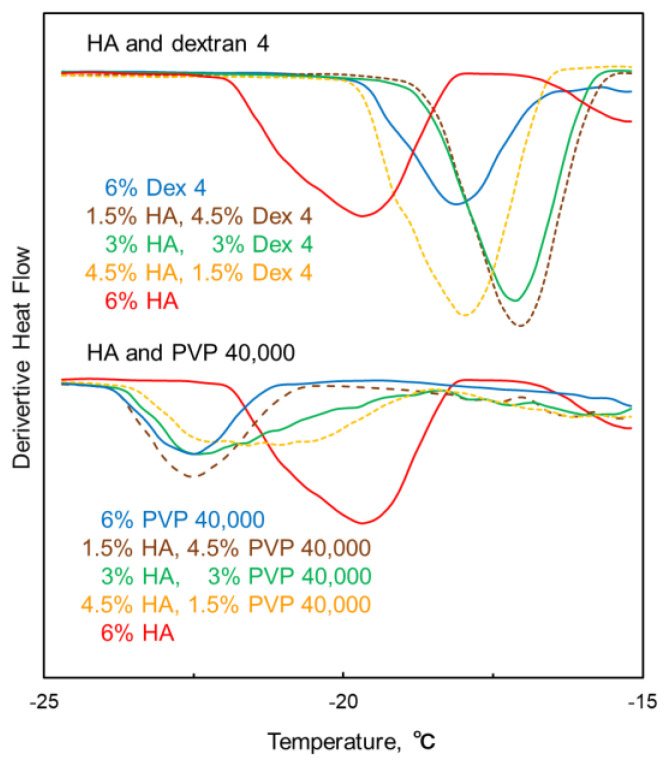
Thermal transitions in the derivative heat flow curves of frozen solutions comprising hyaluronic acid (HA), dextran 4, polyvinylpyrrolidone (PVP) 40,000, and their mixtures.

The close intrinsic T_g_′ of HA and several polymers (temperature margin <5 °C, dextran 4, dextran sulfate 7000–20,000, PVP 29,000, and PVP 40,000) hampered the determination of their mixing states based on the profile of their transition temperatures. However, the shape of the transition in the derivative heat flow curves, largely differed among the polymer combinations. The HA and dextran 4 combination displayed a sharp T_g_′ peak that shifted to higher temperatures than those of the individual components, which strongly suggested the co-operative physical change of the polymers concomitant with some attractive interaction in the freeze-concentrate (Figure 5). Conversely, the broad peaks of the HA and PVP 40,000 combination observed at their equivalent mass ratio suggested the overlapping transitions of the partially separated phases.

### 3.3. Mixing States of HA and Crystallizing Solutes in Frozen Solutions

Mannitol and glycine are typical low-molecular-weight excipients that tend to crystallize during freezing of aqueous solutions, which is often perturbed by the addition of co-solutes [52,53,54]. HA-rich frozen solutions comprising mannitol or glycine exhibited a T_g_′ at temperatures that were lower than that of HA, suggesting mixing of the freeze-concentrated polymer and low-molecular-weight excipients at these mass ratios (Figure 4). Some polymers displayed no apparent peaks or thermal transitions in the single-solute frozen solutions (PVA, sodium alginate, and HEC). The lowering of the transition temperature as the HEC ratio increased suggested the mixing of freeze-concentrated HA and HEC at the HA-rich mass ratios. By contrast, the absence of apparent changes in the transition temperature and shape compared with that of HA in the combination solution suggested the presence of HA and PVA, sodium alginate, or CMC Na in the different phases. PVA tends to crystallize by self-assembly and the formation of a microstructural domain, creating a PVA hydrogel upon freeze-thawing [55,56,57].

**Figure 6:**
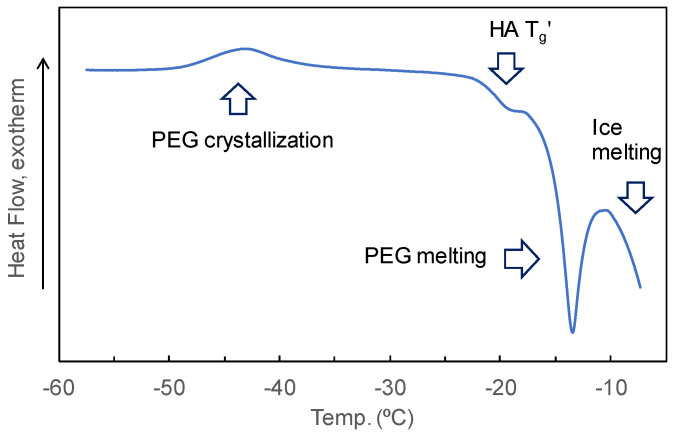
Heat flow curves of frozen solutions comprising 4.5% hyaluronic acid (HA) and 1.5% polyethylene glycol (PEG) 6000 obtained through the heating scan.

**Figure 7:**
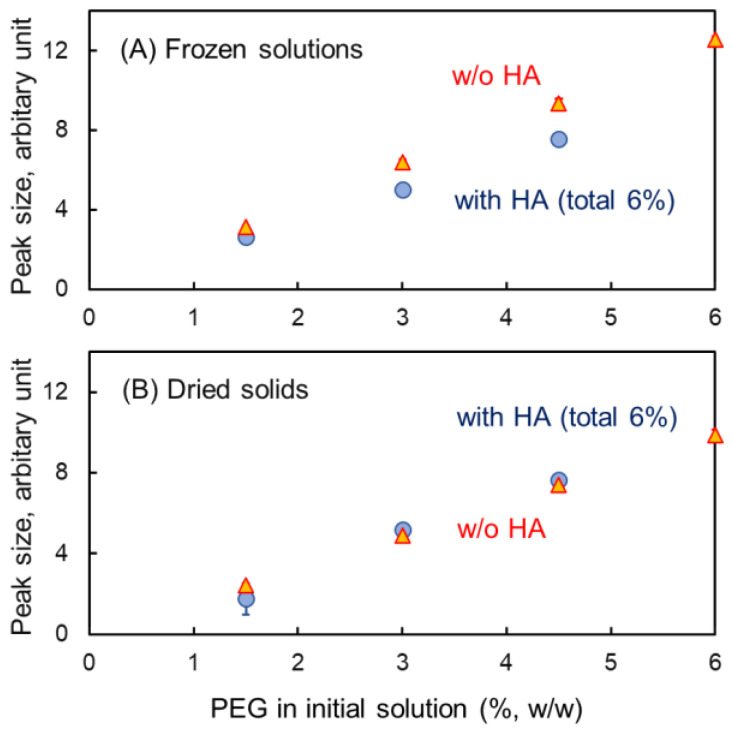
Size of the polyethylene glycol (PEG) melting exotherm peak in the frozen solutions (**B**) and solids dried in the DSC cells (**A**) (n = 3, average ± SD). The symbols denote the peak size of the samples with (◯) and without (△) HA.

Single-solute PEG tends to crystallize and melt in heating scans of single-solute frozen solutions [58]. Certain HA and PEG combinations (e.g., 4.5% HA and 1.5% PEG 6000, *w*/*w*) showed thermal events of the concentrated HA phase (T_g_′ at approx. −20 °C) and PEG phase (crystallization at approx. −43 °C and melting at approx. −14 °C) during a heating scan (Figure 6). The limited effect of HA on the size of the melting exotherm peak of PEG indicated their phase separation at wide mass ratios (Figure 7A). The crystallization and melting peaks should not appear when PEG and other solutes are kept in a mixed state in the freeze-concentrate phase.

### 3.4. Characterization of HA-Based Polymer Films and Microneedles

**Figure 8:**
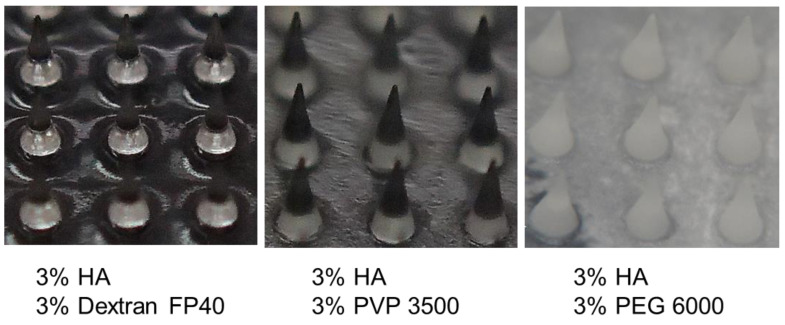
Angled view images of microneedles prepared from aqueous solutions comprising hyaluronic acid (HA) and polymers (6% total) via the micromolding method.

The aqueous polymer solutions comprising HA, polymer, and their combinations were dried at room temperature to prepare films to study the miscibility of the components. Table 3 summarizes the appearance of films obtained by drying aqueous solutions comprising HA and co-solutes (Images in Figure S1). Drying of aqueous HA solutions (6%, *w*/*w*) and HA combined with dextran 4, dextran FP40, gelatin, and HEC (3% each) resulted in transparent films. By contrast, the combination of HA with PVP 3500–40,000, PAA Na, DEAE-dextran 10,000, dextran sulfate Na 5000, PVA 10,000, and sodium alginate (3% each) resulted in slightly cloudy to opalescent films, which suggested the phase separation and/or uneven distribution of the polymers. Drying of the HA and PEG 6000 combination resulted in white brittle solids. A thermal scan of the films revealed the limited effect of HA on the melting peak of the PEG crystal, which appeared at approximately 55 °C (Figure 7B). Among the polymers that showed mixing with HA in frozen solutions, those with DEAE-dextran HCl or dextran sulfate 5000 resulted in the clouding of the film. The preparation of microneedles using the micromolding method resulted in transparent (HA, HA, and dextran), opaque (HA and PVP), and white (HA and PEG) solids (Figure 8), which were essentially similar to those of the films. The tip of needles formed by HA and dextran or PVP appeared darker due to the lighting.

## 4. Discussion

The profiles of the transition temperature (T_g_′) obtained via the thermal analysis of frozen solutions indicated varying mixing states of HA and co-solutes in the concentrated environment. The observed differences in the miscibility were consistent with the reports of the physical properties of concentrated aqueous polymer solutions. PVP, PEG, and PVA are typical polymers that form aqueous two-layer systems with polysaccharides (e.g., dextran) at high concentrations [59,60]. Aqueous two-phase separation of hyaluronic acid and gelatin has also been reported [61]. HA and PEG combinations form an aqueous two-layer system at high concentrations, leading to a particular microstructure in hydrogels [34]. PEG was thought to undergo crystallization as an intrinsic property after the phase separation in the condensed environment [58]. The occurrence of microscopic phase separation of HA and PVA during the preparation of delivery matrices has also been reported [57]. A repulsive interaction between the anionic polymer molecules would explain the observed phase separation in the HA and PAA Na combination [62].

The opaque-to-white films obtained from polymer combination solutions that exhibited phase separation in the frozen solution indicated the relevance of the method to predicting component miscibility in the HA-comprising microneedles and other drug delivery matrices. HA and PVP should be a typical polymer combination that separates into different phases in frozen solutions, films, and microneedles. Air-drying of the 6% PVP solutions resulted in transparent films. The transparent films obtained for the combination of HA and several polymers indicated their mixing in the freeze-concentrate. However, combination with DEAE-dextran 10,000 or dextran sulfate apparently yielded cloudy area in the center of the films. The HA and DEAE-dextran 10,000 molecules likely bind electrostatically in the solution, which would lead to the precipitation of the mixture and the formation of opaque films [50,63]. We must be aware that the observed film haziness does not necessarily indicate phase separation of the polymers. The cause of the film clouding observed in the HA and dextran sulfate combination remains unclear.

Understanding the advantages and limitations of the characterization methods, would assist in the use of the alternative method to characterize the physical properties of multicomponent amorphous pharmaceutical solids [64]. The applicability of the easy-to-handle low-concentration polymer solutions, rapid analysis, and the availability of information on the miscibility of amorphous components in the highly concentrated environment that mimics the air-drying process would be the obvious advantages of characterizing the frozen solutions. Differences in the temperature, processing time, and amount of water should be the major factors that would induce different polymer miscibility in the thermal analysis of frozen solutions and the air-drying of the polymer composites. Drying-related phase separation also occurs in the coating of microneedles [29]. The high temperature and slow air-drying process should provide additional opportunities to induce the inhomogeneous polymer distribution.

This method should provide a better chance to control the physical state of microneedles rationally through formulation and process optimization. The preferred mixing state of the polymers should depend on the objective formulations. The phase-separated solids may be valuable for specific purposes such as providing high physical strength and performance (e.g., the loading and release of active pharmaceutical ingredients (APIs)) in some microneedle formulations [24]. The clear T_g_′ transitions observed in the thermal analysis of many frozen polymer solutions other than HA suggested the applicability of the method for microneedles based on them. Multiple factors that affect polymer interactions (e.g., solution pH and composition of inorganic salts) would have a substantial effect on their mixing state and crystallinity in frozen solutions and dried solids [33]. Methods aimed at detecting and controlling the mixing states of the polymers and APIs represent an interesting topic that requires further study.

## 5. Conclusions

Thermal analysis of the frozen aqueous solutions comprising HA and several polymers (e.g., dextran FP40, DEAE-dextran, dextran sulfate, and gelatin) showed a single T_g_′ transition at temperatures that shifted according to their mass ratio, which indicated the mixing of the freeze-concentrated non-crystalline solutes. By contrast, separation into different phases was strongly suggested in freezing the HA and polyvinylpyrrolidone (PVP) or HA and polyacrylic acid (PAA) combination solutions. The phase separation would explain the observed crystallization of PEG in freezing the solution with HA. The fact that some polymer mixtures, which undergo phase separation in freezing solutions, formed opaque films and microneedles upon drying suggests the usefulness of thermal analysis data in estimating the mixing state of polymers in drug delivery system matrices. We hope that further studies on how the mixing state affects the physical and functional properties of dissolving microneedles will contribute to the rational development of reliable products.

## Figures and Tables

**Table 1:** Glass transition temperatures of maximally freeze-concentrated solutes (T_g_′) in hyaluronic acid (HA) solutions of various molecular weights (MW) and concentrations (n = 3, average ± SD).

Sodium Hyaluronate		Transition Temperature	
Conc.	Name/Size	1st Scan	2nd Scan
% (*w*/*w*)		°C	°C
1	FCH-SU 50–110 kDa	−20.01 ± 0.25	−19.63 ± 0.32
1	FCH-60 500–700 kDa	−20.40 ± 0.15	−19.86 ± 0.17
1	FCH-80 600–1000 kDa	−20.51 ± 0.17	−20.03 ± 0.18
1	FCH-120 1000–1400 kDa	−20.54 ± 0.08	−19.86 ± 0.22
1.5	FCH-SU	−20.15 ± 0.11	−19.64 ± 0.07
3	FCH-SU	−20.31 ± 0.07	−19.65 ± 0.08
4.5	FCH-SU	−20.03 ± 0.08	−19.50 ± 0.14
5	FCH-SU	−19.95 ± 0.06	−19.42 ± 0.08
6	FCH-SU	−19.85 ± 0.17	−19.41 ± 0.12

**Table 2:** Glass transition temperatures of maximally freeze-concentrated single-solute polymer and disaccharide solutions (T_g_′, n = 3, average ± SD).

		Transition Temperature	
Conc.	Name/Size	1st Scan	2nd Scan
% (*w*/*w*)		°C	°C
6	Dextran 4	−18.24 ± 0.09	−18.15 ± 0.12
6	Dextran FP40	−12.06 ± 0.13	−11.84 ± 0.11
6	PVP 3500	−30.27 ± 0.27	−29.81 ± 0.13
6	PVP 10,000	−29.10 ± 0.16	−28.82 ± 0.16
6	PVP 29,000	−22.85 ± 0.12	−22.69 ± 0.06
6	PVP 40,000	−22.40 ± 0.19	−22.13 ± 0.14
6	PAA Na	−36.22 ± 0.35	−35.44 ± 0.23
6	DEAE-dextran 10,000	−36.16 ± 0.07	−35.73 ± 0.04
6	DEAE-dextran HCl	−25.69 ± 0.27	−25.61 ± 0.06
6	Dextran sulfate Na, 7000–20,000	−23.38 ± 0.01	−23.03 ± 0.22
6	Dextran sulfate Na, 5000	−32.05 ± 0.34	−31.64 ± 0.13
6	Gelatin	−9.93 ± 0.21	−9.25 ± 0.11
6	Glucose	−45.65 ± 0.33	−44.34 ± 0.58
6	Sucrose	−34.06 ± 0.12	−33.22 ± 0.31
6	Mannitol	Crystallization	n.d.
6	Glycine	n.d.	n.d.
6	PEG 6000	Crystallization/Melting	Crystallization/Melting
6	Na Alginate	n.d.	n.d.
6	PVA 10,000	n.d.	n.d.
6	CMC Na	n.d.	n.d.
6	HEC	n.d.	n.d.

**Table 3:** Suggested mixing state polymers in frozen solutions and appearance of films obtained by drying aqueous solutions.

Polymers		Miscibility in Frozen Solution *	Film Appearance **
6% HA		M	-
3% HA	3% Dextran 4	M	-
3% HA	3% Dextran FP40	M	-
3% HA	3% PVP 3500	PS	++
3% HA	3% PVP 10,000	PS	++
3% HA	3% PVP 29,000	PS	+
3% HA	3% PVP 40,000	PS	+
3% HA	3% PAA Na	PS	++
3% HA	3% DEAE-dextran 10,000	M	++
3% HA	3% Dextran sulfate Na, 5000	M	++
3% HA	3% Gelatin	M	-
3% HA	3% PEG 6000	PS (crystallized)	+++
3% HA	3% Na Alginate	PS	++
3% HA	3% PVA 10,000	PS	+
3% HA	3% HEC	M	-

* M: mixed, PS: phase-separated; ** -: transparent, +: slight clouding, ++: opalescent, +++: white solid.

## Data Availability

Data are contained within the article.

## Supplementary Materials

The following supporting information can be downloaded at https://www.mdpi.com/article/10.3390/pharmaceutics16101280/s1, Figure S1: Images of films prepared via the air-drying of aqueous solutions comprising hyaluronic acid (HA) and polymers (6% total).
