# Supplementary material for: Application of the Thermal Analysis of Frozen Aqueous Solutions to Assess the Miscibility of Hyaluronic Acid and Polymers Used for Dissolving Microneedles
Source: Pharmaceutics. 2024 Sep 30;16(10):1280. doi: 10.3390/pharmaceutics16101280 (PMC11510125; doi:10.3390/pharmaceutics16101280)
Supplement: Supplementary file 1 [file pharmaceutics-16-01280-s001.zip › pharmaceutics-3204247-supplementary.pdf]

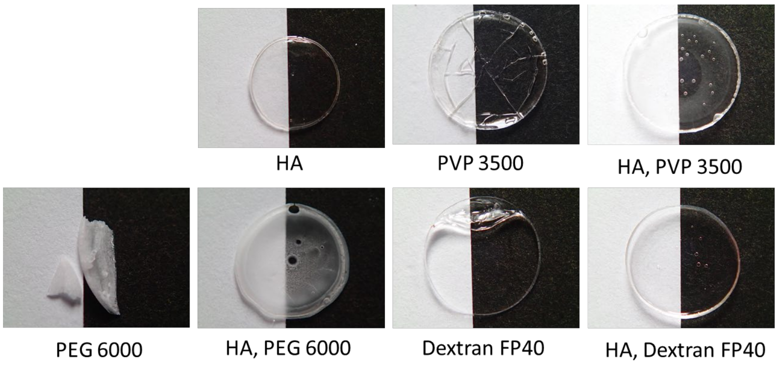

Figure S1. Images of films prepared via the air-drying of aqueous solutions comprising hyaluronic acid (HA) and polymers (6% total).
